# Antibacterial Activity and Mechanisms of Action of Inorganic Nanoparticles against Foodborne Bacterial Pathogens: A Systematic Review

**DOI:** 10.1049/2024/5417924

**Published:** 2024-01-16

**Authors:** Abayeneh Girma, Birhanu Abera, Bawoke Mekuye, Gedefaw Mebratie

**Affiliations:** ^1^Department of Biology, College of Natural and Computational Science, Mekdela Amba University, P.O. Box 32, Tuluawlia, Ethiopia; ^2^Department of Physics, College of Natural and Computational Science, Mekdela Amba University, P.O. Box 32, Tuluawlia, Ethiopia

## Abstract

Foodborne disease outbreaks due to bacterial pathogens and their toxins have become a serious concern for global public health and security. Finding novel antibacterial agents with unique mechanisms of action against the current spoilage and foodborne bacterial pathogens is a central strategy to overcome antibiotic resistance. This study examined the antibacterial activities and mechanisms of action of inorganic nanoparticles (NPs) against foodborne bacterial pathogens. The articles written in English were recovered from registers and databases (PubMed, ScienceDirect, Web of Science, Google Scholar, and Directory of Open Access Journals) and other sources (websites, organizations, and citation searching). “Nanoparticles,” “Inorganic Nanoparticles,” “Metal Nanoparticles,” “Metal–Oxide Nanoparticles,” “Antimicrobial Activity,” “Antibacterial Activity,” “Foodborne Bacterial Pathogens,” “Mechanisms of Action,” and “Foodborne Diseases” were the search terms used to retrieve the articles. The PRISMA-2020 checklist was applied for the article search strategy, article selection, data extraction, and result reporting for the review process. A total of 27 original research articles were included from a total of 3,575 articles obtained from the different search strategies. All studies demonstrated the antibacterial effectiveness of inorganic NPs and highlighted their different mechanisms of action against foodborne bacterial pathogens. In the present study, small-sized, spherical-shaped, engineered, capped, low-dissolution with water, high-concentration NPs, and in Gram-negative bacterial types had high antibacterial activity as compared to their counterparts. Cell wall interaction and membrane penetration, reactive oxygen species production, DNA damage, and protein synthesis inhibition were some of the generalized mechanisms recognized in the current study. Therefore, this study recommends the proper use of nontoxic inorganic nanoparticle products for food processing industries to ensure the quality and safety of food while minimizing antibiotic resistance among foodborne bacterial pathogens.

## 1. Introduction

Pathogenic and spoilage-causing agents must be controlled in a variety of foods to ensure food quality and safety [1–4]. The sporadic prevalence of microbial pathogens in food and the increased incidence of antibiotic-resistant strains and their genes have posed serious concerns for public health. Currently, modern food processing is also confronted with a challenge due to pathogenic and spoilage microbes or their toxins resistance in foods, resulting in huge economic losses [5–7]. To overcome these urgent problems, novel agents with unique mechanisms of action are needed for effective control of current bacterial pathogens in food and the environment. This has sparked a lot of interest in so-called “nanotechnology,” an emerging area such as the technology of production, characterization, and application of materials at the nanoscale.

Nanotechnology is now entering the food processing industry to preserve and prolong the shelf life of foods and to decontaminate and control spoilage and foodborne microbial pathogens [8]. This is due to the outstanding properties of nanoparticles (NPs), such as biocompatibility, high productivity, speed of production, cost-effectiveness, and safety [9]. Furthermore, due to their exceptional and novel properties, such as their small particle size and high surface area, NPs with diameters smaller than 100 nm demonstrate a wide spectrum of effective antibacterial activities [10]. The positive charge on the surface of the NPs increases their attachment to the negatively charged membrane surface of the bacterium, which may boost the antibacterial impact [11]. It has been demonstrated that AgNPs with a spherical shape are more efficient against bacteria than those with a rod-like structure [12]. Higher NP concentrations imply more ions, which results in intensive contact with bacterial cells, leading to higher antibacterial activity [13]. Capping agents have a substantial impact on the antibacterial activity of AgNPs [14]. The effects of NPs on Gram-positive and Gram-negative bacterial species varied due to variations in bacterial cell wall structure. According to numerous studies, NPs have better antibacterial effects on Gram-negative bacteria strains than they do on Gram-positive bacteria [15]. The origin of NPs can also affect their antibacterial activity.

In recent years, metal and metal oxide-based inorganic NPs such as silver (Ag) [16], gold (Au) [17], selenium (Se) [18], zinc oxide (ZnO) [19], magnesium oxide (MgO) [20], and iron oxide (IO) [21] have been recognized to display antibacterial activity; however, there is an ongoing debate regarding their antibacterial mechanisms. Even though Tsikourkitoudi et al. [22] demonstrated that inorganic nanoparticles primarily exert antimicrobial activity through metal-ion release, reactive oxygen species (ROS)-induced oxidative stress, as well as nonoxidative mechanisms, these mechanisms may include disruption of the microbial cell wall or membrane, oxidation and/or damage of microbial cell components, DNA damage, and interruption of electron-transport processes.

Indeed, as per our knowledge, the antibacterial activities and mechanisms of inorganic NPs against foodborne bacterial pathogens have not been touched, collected, organized, or presented as a systematic review. Therefore, this study was intended to address this issue using previously published works considering the antibacterial activity of various inorganic NPs and mechanisms of action against foodborne bacterial pathogens and provides valuable information to researchers, food processing industries, stalk holders, and the general public.

## 2. Methodology

Several research articles on NPs for controlling foodborne bacterial pathogens were extensively searched and collected in different databases. Many published articles were available separately, and a detailed review was essential to combine all the results to draw a conclusion and avoid any information conflicts, ambiguities, or misunderstandings. The review, which aims to highlight the method of synthesis, the characterization method, the particle size, antibacterial activities, antibacterial mechanisms, and the main result, was conducted according to systematic reviews, as recommended by Page et al. [23]. The PRISMA-2020 (i.e., Preferred Reporting Items for Systematic Reviews and Meta-Analyses) guideline and checklist were strictly followed to document this review.

### 2.1. Formulation of Research Questions and Problems

This systematic review was guided by the question, “What are the antibacterial activities and mechanisms of action of inorganic nanoparticles in controlling foodborne bacterial pathogens?” The problem was formulated during searching and assessing the importance of nanoparticles to the current world in varying fields of study. As a result of their diverse significance, the study concentrates on examining the antibacterial activity of inorganic NPs. This question further interests us in examining whether inorganic NPs could serve as an absolute option to combat foodborne bacterial pathogens using unique mechanisms of action and replace the existing pharmaceutical drugs or whether these inorganic NPs could be used as alternative options.

### 2.2. Search Engine for Research Articles

**Figure 1:**
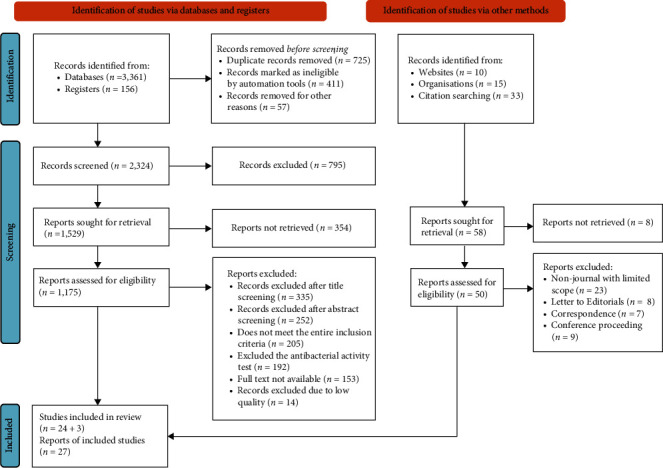
PRISMA-2020 flow diagram of eligible studies.

An extensive search of research articles was conducted in registers and databases (PubMed, ScienceDirect, Web of Science, Google Scholar, and Directory of Open Access Journals) and other sources (websites, organizations, and citation searching). The research articles were searched using the following key terms and phrases taken from the title, abstract, and keywords in combination or separately using Boolean operators (“OR” or “AND”): “Nanoparticles,” “Inorganic Nanoparticles,” “Metal Nanoparticles,” “Metal–Oxide Nanoparticles,” “Antimicrobial Activity,” “Antibacterial Activity,” “Foodborne Bacterial Pathogens,” “Mechanisms of Action,” and “Foodborne Diseases.” The study was carried out from September 2022 to March 2023. The search process was presented according to the PRISMA-2020 flow diagram guidelines [23], together with the included and excluded items and reasons for exclusion (Figure 1).

### 2.3. Inclusion and Exclusion Criteria for Included Studies

#### 2.3.1. Inclusion Criteria

- Original articles on inorganic nanoparticles that address antibacterial activities against foodborne bacterial pathogens.
- Experimental study design.
- Only bacterial causative agents.
- Recent studies reported in English and available online were included in this study.

#### 2.3.2. Exclusion Criteria

- Reports on other nanoparticles and the role of food packaging alone or not linked to antibacterial-dependent results.
- Other causative agents (fungi, parasites, viruses).
- Studies not peer-reviewed and published in other languages.
- Previously reviewed papers, low-quality articles, and duplicate publications or extensions of analysis from original studies.

### 2.4. Data Extraction

A data abstraction protocol was used to construct data from each of the included articles. The data extraction protocol consists of the type of inorganic nanoparticles, method of synthesis, characterization, particle size, foodborne bacterial pathogens (Gram-positive and Gram-negative), antibacterial mechanisms, main result, and references for Table 1. Furthermore, in Table 2, we used the type and source of inorganic nanoparticles, foodborne bacterial pathogens, concentration, and antibacterial efficacy tests of inorganic nanoparticles (MIC, MBC, and ZOI), the main factors influencing efficacy, and references. The selection of all retrieved articles was carried out step by step by two independent groups (AG and BA) and (BM and GM), and finally, the extracted data were combined and clearly presented in the table with the key information and findings.

### 2.5. Quality Assessment of Each Included Studies

The PRISMA-2020 checklist item is the best tool to assess the validity, reliability, and presentation quality of all data from the included articles as a systematic review [23]. The Grading of Recommendations Assessment, Development, and Evaluation approach was used to evaluate the overall quality of the evidence. The quality of each study was assessed using the three primary assessment criteria (methodological quality, comparability, study outcome, and statistical analysis) [51]. Publications of high quality were awarded five to six points, those of moderate quality four points, and articles of low quality zero to three points. The choice and evaluation of the quality were performed independently by the four reviewers (AG, BA, BM, and GM). The articles were added after agreement was reached, and the discrepancies between the reviewers were resolved through discussion.

## 3. Results

### 3.1. Outcome of the Literature Search

**Figure 2:**
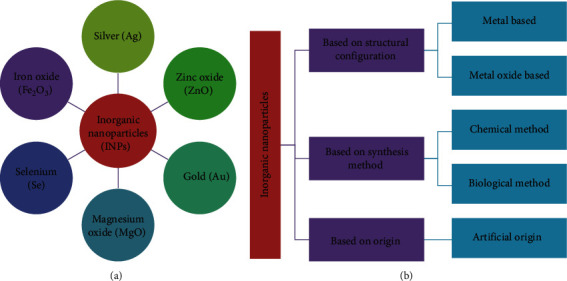
(a) Inorganic NPs. (b) Classification of inorganic NPs according to the included studies.

**Figure 3:**
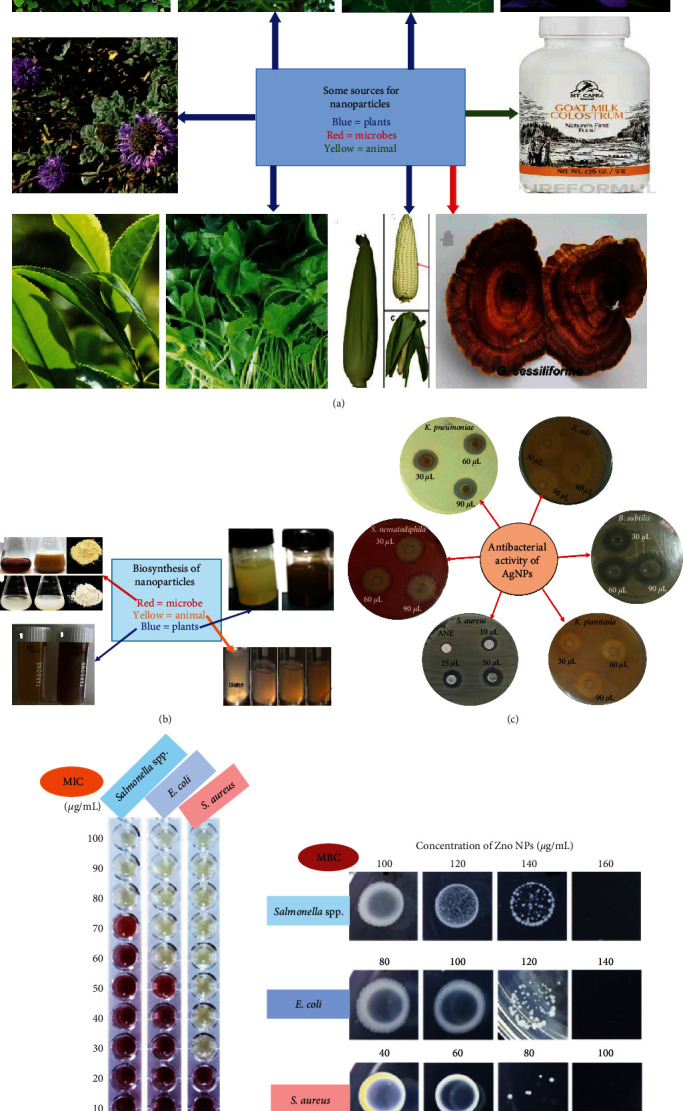
(a) Sources of NP. (b) Synthesis of NPs from different natural sources. (c) Antibacterial activity test of AgNPs against different foodborne bacterial pathogens. (d) MIC and MBC test of ZnONPs against three foodborne bacterial pathogens.

**Figure 4:**
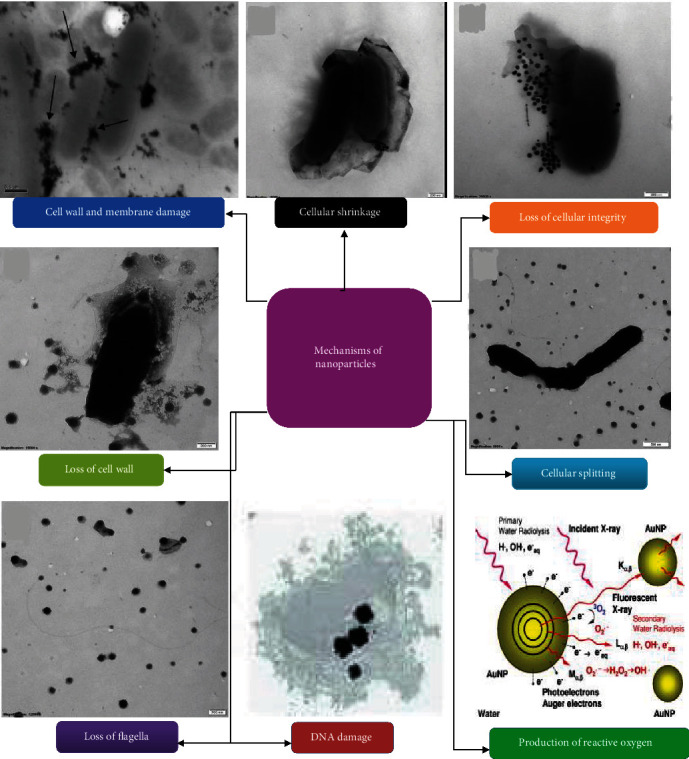
Mechanisms of action of inorganic nanoparticles are demonstrated in the current study.

**Figure 5:**
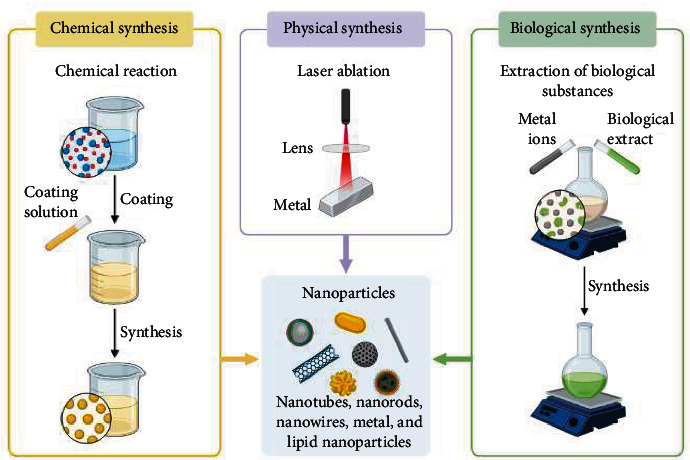
Nanoparticle synthesis methods in laboratory (created by BioRender program https://biorender.com/).

**Figure 6:**
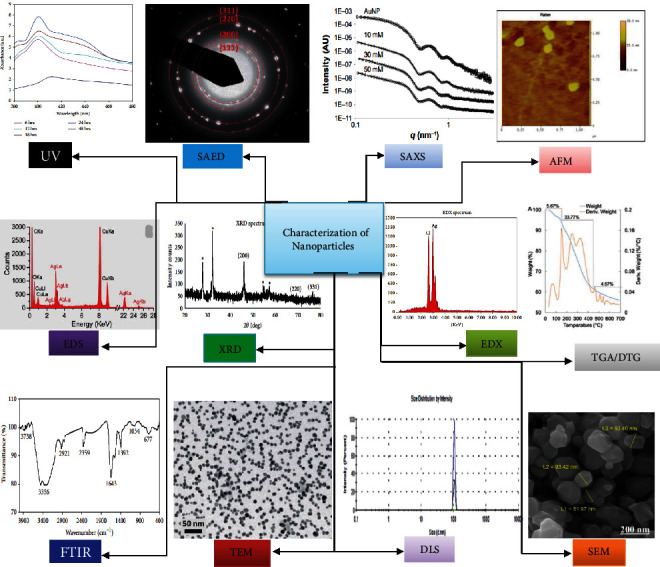
Different characterization methods of inorganic nanoparticles were used in the study.

All included studies were conducted on inorganic NPs (Figure 2) and showed antibacterial activities against foodborne bacterial pathogens (Figure 3(c), Tables 1 and 2), of which the majority demonstrated antibacterial mechanisms (Table 1 and Figure 4). All included studies used artificial-origin NPs (Figures 3(a) and 3(b), and Table 2). Articles included in the present study were synthesized by bottom-up approaches using chemical or biological synthetic methods (Figures 3(b) and 5, Table 1) and. UV, SAED, SAXS, FAM, EDS, XRD, EDX, TGA/DTG, FTIR, TEM, DLS, and SEM (Figure 6 and Table 1) were used by investigators to characterize its nanoparticles. Different nanoparticles demonstrated different antibacterial activities at different concentrations (Figure 3(c) and Table 2). Disc diffusion, well diffusion, MIC, and MBC tests were used to check the antibacterial effectiveness of inorganic nanoparticles in the laboratory (Figure 3(d) and Table 2), and factors that affect their antibacterial effectiveness are presented in Tables 2 and 3.

### 3.2. General Characteristics of the Eligible Studies

In total, 3,575 articles on the use of nanoparticles in preventing or controlling foodborne bacterial pathogens were recovered throughout the world. In total, 1,193 records were removed before screening (duplicate records removed (*n* = 725), records marked as ineligible by automation tools (*n* = 411), and records removed for other reasons (*n* = 57)). Of the remaining 2,324 articles, 795 were further excluded. Of the remaining 1,587 articles in registers, databases, and other methods, 362 were not retrieved. Of 1,225 articles, 1,198 were further excluded after observation and review due to the inclusion and exclusion criteria used. Only 27 reports were included in the final analysis (Figure 1). Among the 27 articles, 13, 5, 4, 2, 2, and 1 examined, respectively, the antibacterial activities of silver, zinc oxide, gold, selenium, magnesium oxide, and iron oxide NPs (Table 1). Of the included articles, 20 investigated the antibacterial mechanisms of NPs, while the remaining seven articles did not address them (Table 1). Nine and 18 studies used chemically and biologically synthesized nanoparticles, respectively (Table 1). Twenty-three of the included articles were evaluated for their NP activity against Gram-negative and Gram-positive foodborne bacterial pathogens, and the other four articles were assessed for Gram-negative bacteria only (Table 1). Twenty-four of the included articles investigated the particle size of NPs, and in the remaining three articles, the NP sizes were unknown (Table 1).

## 4. Discussion

Foodborne diseases are major public health concerns that cause morbidity and mortality across the globe [59]. Various antimicrobial agents are still applicable in the food industry to preserve and decontaminate foods and food products, as well as to destroy bacterial agents [60]. However, some of these antimicrobials are resistant to various foodborne bacterial pathogens [7]. Therefore, developing new agents with alternative mechanisms of action against the current foodborne bacterial pathogens is crucially needed. Currently, inorganic NPs (silver, zinc oxide, gold, selenium, magnesium oxide, and iron oxide) are being increasingly studied for their antibacterial properties and potential applications in biomedicine [61] and the food industry [62], along with minimizing treatment durations, side effects, and antimicrobial resistance [63]. Knowing alternative antimicrobial agents and their unique mechanisms of action against potential foodborne bacterial pathogens and their toxins is useful as a guide for both governmental and nongovernmental policymakers and stakeholders to control food-related diseases.

### 4.1. Antibacterial Activities of Inorganic Nanoparticles

In this study, inorganic NPs showed significant antibacterial activities against both Gram-positive and Gram-negative foodborne bacterial pathogens. Rajeshkumar and Malarkodi [25], Nam et al. [27], Patra and Baek [28], Zarei et al. [34], Mohanta et al. [35], Saratale et al. [36], Alelwani et al. [39], Eze et al. [40], Chauhan et al. [41], Loo et al. [42], Khorasani et al. [43], El-Batal et al. [45], and Kanmani and Lim [48] demonstrated the antibacterial activities of silver nanoparticles (AgNPs). Similarly, the antibacterial activities of AgNPs were reported elsewhere by Rhim et al. [64], Balachandar et al. [65], and Alsammarraie et al. [66]. However, Yusof et al. [26], Ali et al. [32], Pawar et al. [44], Morsy et al. [47], and Tayel et al. [50] showed the antibacterial effectiveness of zinc oxide nanoparticles (ZnO-NPs). Similarly, de Souza et al. [19], Xie et al. [67], and Liu et al. [68] reported the effectiveness of ZnO-NP against foodborne bacterial pathogens. Additionally, Zawrah et al. [24], Rattanata et al. [29], Chandran et al. [30], and Hameed et al. [33] showed the antibacterial activities of gold nanoparticles (AuNPs). Su et al. [69], Lee and Lee [70], and Hatipoğlu and Rubinstein [71] mutually support the antibacterial effectiveness of AuNPs. Differently, Jin and He [37] and He et al. [49] demonstrated the antibacterial activities of magnesium oxide nanoparticles (MgO-NPs). Agreeably, Imani and Safaei [20], Nguyen et al. [72], and Maji et al. [73] reported the antibacterial properties of MgO-NPs. Furthermore, the antibacterial activities of selenium nanoparticles (SeNPs) were demonstrated by Khiralla and El-Deeb [31], Meenambigai et al. [38], and Iron oxide nanoparticles (IO-NPs) by Heidari et al. [46]. Similar reports have been presented elsewhere by Alghuthaymi et al. [74], ElSaied et al. [75], and Hernández-Díaz et al. [76] for SeNPs and Mohan and Mala [77] and Bankole et al. [78] for IO-NPs, respectively.

### 4.2. Factors Affecting the Antibacterial Activities of Inorganic Nanoparticles

In this study, the antibacterial activities of different inorganic NPs were reported to be influenced by various factors such as the type of bacterial species, particle size, shape, charge, concentration, and type of capping or stabilizing agents used (Table 3). Consistently, Kim et al. [79], Zhang et al. [80], Pal et al. [81], Meire et al. [82], Martínez-Castañon et al. [83], Fayaz et al. [84], and Singh et al. [85] reported that the type of bacteria, concentration, shape, and size, as well as the combination of different antibiotics, are the parameters that can alter the bactericidal activity of AgNPs. Furthermore, the stability, size, and morphological characteristics of nanoparticles are influenced by a variety of variables, including the synthetic method, solvent, temperature, concentration, and strength of the reducing agent [86].

Regarding the bacterial Gram-type, the antibacterial activities of NPs were higher among Gram-negative bacteria than Gram-positives. Similarly, Priyadarshini et al. [87] reported that *Escherichia coli*, a Gram-negative bacterium, showed a greater zone of inhibition (ZOI) compared to *Bacillus cereus* and *Streptococcus pyogenes*, which are Gram-positives. A greater growth inhibition zone was observed for the Gram-negative bacterium *Pseudomonas aeruginosa* (15 mm), followed by the Gram-positive *Staphylococcus aureus* (14 mm). Previously, Kim et al. [79] also reported that the Gram-negative bacteria *E. coli* was more susceptible to AgNPs than the Gram-positive *S. aureus* bacteria. This might be due to the fact that the cell walls of Gram-positive bacteria are composed of ∼20–80 nm peptidoglycan, which is comparatively thicker than the ∼7–8 nm peptidoglycan found in Gram-negative bacteria. Furthermore, Gram-positive bacteria have a more complex peptidoglycan coating than Gram-negative bacteria, which makes it more difficult for NPs to penetrate them. Since this layer contains linear polysaccharide chains and is cross-connected by more short peptides [87].

With respect to size, small-sized NPs have the greatest antimicrobial effect in comparison to larger ones because of their innovative tiny size and increasing surface-to-volume ratio. According to Duncan [88], the size and shape may influence how effective they are against pathogenic microbes. AgNPs with 1–10 nm particle size have previously been reported to show the most effective antibacterial activities through direct interaction with the cell wall and membranes of bacteria, causing pits and holes to form, sugar reduction leakage, and ultimately bacterial death [89, 90]. The antibacterial effect of NPs increases considerably with decreasing size, which may be because smaller particles have more surface area for releasing silver ions and also have greater protein binding capabilities. Additionally, tiny particles can easily flow through the pores in the bacterial membrane and readily reach the bacteria.

Regarding the shape of NPs, Dakal et al. [91] demonstrated that the potential antimicrobial effect changes with the shape of NPs. Furthermore, Raza et al. [92] and Acharya et al. [93] also confirmed that the interaction of AgNPs with bacteria is shape-dependent. Cheon et al. [94] demonstrated the antibacterial activity of AgNPs in three different shapes—spherical, disc, and triangular—and reported that the highest bactericidal effect was observed against spherical, which is greater than disc, and disc, which is greater than triangular, AgNPs. Spherical AgNPs showed the most significant antibacterial activity against *E. coli* and *Bacillus* bacteria, according to research by Ashkarran et al. [95] that looked at the antimicrobial activity of four different shaped silver nanostructures (wiry, cubic, spherical, and triangular) against *Staphylococcus*, *Bacillus*, and *E. coli* bacteria. Laha et al. [96] also showed that CuO-NPs with a spherical shape had greater antibacterial activity on Gram-negative bacteria. ZnO-NPs with sizes of 84 and 27 nm were created by Rajiv et al. [97] and tested for their antifungal effects on *Aspergillus niger*, and it was discovered that hexagonal ZnO-NPs with a diameter of 84 nm are less effective at inhibiting the growth of fungi than spherical ZnO-NPs with a diameter of 27 nm. In another study [54], it has also been reported that the surface-volume ratio is increased as the NPs become smaller and more spherical, which enhances their chemical and biological activity more than nonspherical structures (e.g., rod, discoid, cylinder, etc.). This might be due to the fact that silver ions are reactive due to their facets (111) and (100) [98]. However, only facet (111), which is found in high percentages in spherical-shaped particles, has a high atomic density, which improves the ability of Ag to bind to components that contain sulfur in bacteria and accelerates its death.

With respect to charge, a few investigations have demonstrated that the bactericidal effects of this compound may be caused by the electrostatic interaction between positively charged nanoparticles and negatively charged bacterial cells [99]. Additionally, it is thought that NPs with positive charges interact with negative charges on bacterial cell membranes, disrupting their cell walls and surface proteins and ultimately causing cell death [100, 101]. Also, the antimicrobial effect of NPs having a positive charge on the surface is also high, hence their binding affinity to the negatively charged bacteria cell. For example, it has been suggested that Ag+ primarily exerts antibacterial activity through different modes of action, such as denaturing the 30 s ribosomal component and inhibiting the synthesis of proteins and enzymes necessary for the production of ATP [81, 102]. It also inhibits respiratory enzymes by increasing the formation of ROS [103]. This could be due to the silver ions that AgNPs inject into the bacterial cells, increasing their bactericidal activity [104]. For the microorganisms, *P. aeruginosa* and *S. aureus*, Song et al. [105] demonstrated plasmolysis and inhibition of the formation of the bacterial cell wall by AgNPs.

In the current study, the ZOI increased with increasing NP concentrations against tested bacterial pathogens. This is in agreement with the reports of Song et al. [105]. It demonstrates that the ability of NPs to interact with the cell walls of bacteria decreases at extremely low concentrations, while high quantities enhance the interactivity and bactericidal effects. Liu et al. [68] have previously stated that the bactericidal property of ZnO-NPs depends on the concentration and size of nanoparticles. According to Liu et al. [68] finding, ZnO-NP exhibited effective antibacterial properties against the most important foodborne pathogen, enterohemorrhagic *E. coli* (EHEC) O157:H7, and the bactericidal effects increased as the concentrations of ZnO-NP increased. This is due to the fact that a larger concentration of NPs releases more metal ions, which in turn increase cellular oxidative stress, producing higher antibacterial activity through diffusing into the agar than their counterparts (low concentrations).

Regarding dissolution, the dissolution of NPs plays a crucial role in their antibacterial activity. Different types of NPs exhibit varying dissolution properties. For instance, CuO-NPs (20 nm) in ultra-pure water dissolve up to 95% at a pH value of 5.5 [106]. Similarly, AgNPs (80 nm) for natural river water dissolve up to 3% after just 6 hr in Tween-AgNPs and a similar level in 15 days in citrate and bare-Ag NPs [107], and ZnO-NPs (20–30 nm) for natural seawater dissolve up to 32% at an initial concentration of 10 mg/L [108]. AgNPs [109] and ZnO-NPs also reported low-dissolution activity in water. This may be due to the fact that NPs with smaller sizes have greater specific surface areas, higher surface energies, stronger intermolecular forces, and thus less stable dispersion [109]. The dissolution of nanoparticles is affected by several factors, including particle size, media pH, the presence of dissolved organic material, electrolytes, and capping agents [110, 111]. Shape and surface morphology are two other characteristics that may cause large variations in surface area and alter particle solubility. Particles with a smaller radius of positive curvature (convex) and convex surface properties are more energetically unstable, allowing for preferential dissolving and higher equilibrium solubility. The stability and dissolution of NPs are critical parameters in determining their toxicity and fate in the environment. Zn^2+^ ions and Cu^2+^ ions are released into water systems by ZnO-NPs and CuO-NPs, resulting in toxicity against living organisms [112, 113]. This might be due to the fact that nanoparticle ions (e.g., titanium, silver, and zinc) generate free radicals and lead to the induction of oxidative stress (i.e., ROS) compared to particulate form, which enhances the antimicrobial activities against disease-causing agents [114, 115]. Further, ionic nanoparticles, compared to particulate or particle forms, can often exhibit enhanced efficiency due to several factors, such as an increase in surface area, improved reactivity, enhanced stability, tailorable properties, and unique optical and electrical properties. Ionic nanoparticles have a higher surface area compared to larger particles. This property is particularly beneficial in fields like catalysis, where higher reactivity leads to better conversion rates and faster reactions [116].

In the present study, Du et al. [117] biosynthesized AgNPs against foodborne bacterial pathogens and confirmed that, in comparison to Gram-negative bacteria, Gram-positive bacteria had higher MIC and MBC values. According to Du et al. [117], *Vibrio parahaemolyticus* and *S. aureus* had MICs of 6.25 and 50 g/mL, respectively, whereas their MBCs were 12.5 and 100 g/mL, respectively. Similarly, Nam et al. [27] discussed that the MIC of AgNPs for S. aureus, a Gram-positive bacterium, was twice larger than that of *P. aeruginosa*, *S. enterica*, and *E. coli*, which are Gram-negatives. Further, according to Zarei et al. [34], the three foodborne pathogens tested that were Gram-negative (*E. coli* O157:H7, Salmonella Typhimurium, and *V. parahaemolyticus*) had MIC values of 3.12 g/mL, while *Listeria monocytogenes* displayed a value of 6.25 g/mL. This implies that the MIC value depends on the type of bacteria that are exposed to it. This might be due to the fact that, during bactericidal activity, Gram-positive bacteria with more peptidoglycan cellular layers were more resistant to the penetration of nanoparticles into the cytoplasm than Gram-negative bacteria were [118]. Gram-negative bacteria's significantly thinner cell walls made it possible for two mechanisms to take place: (i) interaction with silver ions and (ii) nanomechanical assaults, which reduced their MICs. The fact that the MICs of silver nanoparticles are lower than those of silver ions is also due to the combination of these two biocidal effects of silver nanoparticles [119, 120]. The MICs of AgNPs, for instance, were 7.8 and 31.2 mg/mL for *E. coli* and *S. aureus*, while the MICs of silver ions were 15.6 and 62.5 mg/mL for each [120]. According to the study by Erjaee et al. [120], *E. coli* had the lowest MIC of the Gram-negative microorganisms, indicating the highest potential for cleaning and sanitizing food-related settings.

### 4.3. Antibacterial Mechanisms of Inorganic Nanoparticles

Inorganic NPs have three primary antibacterial effects: (i) cell wall interaction and membrane penetration; (ii) ROS production; and (iii) DNA damage and protein synthesis inhibition were some of the generalized mechanisms recognized in the current study. Similar to the present study, Häffner and Malmsten [121] reported that, in addition to directly disrupting membranes, nanoparticles can also cause oxidation-sensitive lipids and proteins to be damaged through the production of ROS, damage DNA, impair the functionality of cellular proteins and enzymes, cause inflammation, and impair mitochondrial function. According to Khezerlou et al. [115], mechanisms through which NPs fight infections with antibacterial action include ROS, which are caused by oxidative stress and are induced as a result of free radical production by NPs and their ions (such as those from titanium, silver, and zinc). The pathogens' cellular components, such as their membrane, DNA, proteins, and mitochondria, can be irreversibly damaged and destroyed by the produced ROS, leading to cell death. Similar to the present study, Dakal et al. [91] also reported that the following are some of the ways that metallic nanoparticles work: (i) attraction to bacterial cell walls due to opposite surface charges; (ii) membrane instability; (iii) production of ROS; (iv) release of metal ions; and (v) modification of the signaling pathway.

#### 4.3.1. Interaction with Cellular (Cell Wall and Membrane) Compartments

Nanoparticles cling to cell walls and membranes after being exposed to microbes. The NPs' positive surface charge is essential for attachment. The negatively charged cell membrane of the microorganisms and the positively charged nanoparticles are electrostatically attracted to one another, making NP adhesion to cell membranes easier since they are positively charged in water [122, 123]. Upon such interaction, morphological changes become obvious and can be distinguished by cytoplasmic shrinkage and membrane detachment, which ultimately result in cell wall rupture [67, 124]. According to transmission electron microscopy, the cell membrane of *E. coli* cells totally ruptures after a short period of contact with AgNPs. When AgNPs cause damage, the cell wall becomes circumferential, and TEM images show multiple electron-dense pits at those locations. For the microorganisms, *P. aeruginosa* and *S. aureus*, Song et al. [105] demonstrated plasmolysis and inhibition of the formation of the bacterial cell wall by AgNPs. In addition to electrostatic attraction, the interaction of NPs with the proteins in the cell wall that contain sulfur results in irreversible changes in the cell wall structure, which causes its destruction [125]. This, in turn, has an impact on the cell membrane's permeability and lipid bilayer integrity. Increased membrane permeability as a result of morphological changes in cells has an impact on their capacity to control transport activities through the plasma membrane. The transport and release of potassium (K^+^) ions from microbial cells can also be affected by metal ions. Similarly, a study found that superparamagnetic iron oxide interacts with microbial cells by directly penetrating the cell membrane and interfering with the transmission of transmembrane electrons. The increase in membrane permeability may have more severe repercussions than just impairing transport function, such as the loss of cellular contents through leakage, like ions, proteins, reducing sugars, and occasionally the cellular energy reserve, ATP [79, 90, 91, 126].

#### 4.3.2. Binding to Proteins

The alternative antibacterial mechanisms exhibited by inorganic NPs are protein dysfunction and enzyme inactivation. For example, it has been suggested that Ag+ primarily exerts antibacterial activity through different modes of action, such as denaturing the 30 s ribosomal component and inhibiting the synthesis of proteins and enzymes necessary for the production of ATP via oxidation of amino acid side chains [81, 102]. For instance, protein deactivation results from persistent SAAg bonds formed when the Ag (+) ion connects to thiol groups of proteins present in the cell membrane [127]. AgNPs and Ag (+) ions interact with proteins to change their 3D structure, disrupt disulfide bonds, and block active binding sites, which causes general functional problems in the microorganism [126]. Furthermore, inhibition of phosphorylation of proteins would inhibit their enzymatic activity, which in turn would result in inhibition of bacterial growth. Similarly, studies including the inactivation of cellular proteins, DNA damage, and disruption of metabolic enzymes can be implicated in the beneficial antimicrobial activities of inorganic NPs [85, 98, 128–133]. This might be due to the fact that NPs have a significant potential to inactivate common activities or metabolic processes, such as permeability, respiration, and energy generation, in bacterial pathogens.

#### 4.3.3. Formation of Reactive Oxygen Species

Regarding ROS, it is thought that the inorganic NPs could enter the bacterium and inactivate the respiratory enzymes by accelerating the production of free radical species such as hydrogen peroxide (H_2_O_2_), superoxide anion (O_2_^−^), hydroxyl radical (HO.), hypochlorous acid (HOCl), and singlet oxygen (^1^O_2_), which ultimately results in bacterial death [134]. According to Yu et al. [135], the excessive ROS produced by nanoparticles can damage biomolecules and organelle structures because of their high oxidation potential. This damage includes protein oxidative carbonylation, lipid peroxidation, DNA/RNA breakage, and membrane structure destruction, all of which can result in necrosis, apoptosis, or even mutagenesis. Moreover, ROS are beneficial for increasing the gene expression levels of oxidative proteins, which is a key mechanism in bacterial cell apoptosis. For example, the hydroxyl radical (OH.), one of the most potent radicals, is known to react with all components of DNA, causing single-strand breakage via the formation of an 8-hydroxyl-2′-deoxyguanosine (8-OHdG) DNA adduct [136, 137]. This could be due to the silver ions that AgNPs inject into the bacterial cells, increasing their bactericidal activity [104]. It has also been suggested that AgNPs specifically target and disrupt the respiratory chain by interacting with the thiol groups found in enzymes like NADH dehydrogenases, ultimately causing cell death [85]. As a result, it is anticipated that increasing levels of Ag (+) ions may enhance oxidative stress, which has both cytotoxic and genotoxic effects. The rise of cellular oxidative stress in microorganisms is a sign of the harmful effects of heavy metal ions like Ag (+). This toxic effect may be due to the binding of Ag (+) ions onto the cell membrane of the microbes, which consequently relays signaling and blocks the respiratory function of the microbes. The Ag (+) ion is known to cause dysfunction in the respiratory electron transport chain by uncoupling it from oxidative phosphorylation and inhibiting respiratory chain enzymes.

#### 4.3.4. Interaction with DNA

Microbial cells exposed to NPs also undergo genomic alterations, such as condensation of genetic materials, particularly genomic and plasmid DNA. As a consequence, various important cellular functions get suppressed, which ultimately leads to cell necrosis and death. According to Rai et al. [103], NPs have a high affinity for interaction with substances containing sulfur and phosphorus, such as DNA and proteins on bacterial cell membranes, alter membrane permeability, damage the respiratory chain and cell division machinery, and ultimately cause cell death.

Furthermore, the interaction of AgNPs with DNA may result in DNA shearing or denaturation as well as a disruption of cell division [138, 139]. NP-induced genotoxicity includes chromosomal aberrations such as mutations, DNA strand breaks, and oxidative DNA base damage. In *E. coli*, AgNPs result in DNA damage (such as strand breaks) and mutations in crucial DNA repair genes (mutY, mutS, mutM, mutT, and nth), rendering mutant strains more vulnerable to AgNP-based antimicrobial treatment than wild-type strains [140]. The H-bonds between base pairs of the anti-parallel DNA strands are broken when the Ag (+) ion intercalates between purine and pyrimidine base pairs, causing the double helical shape to be broken [141]. In microorganisms, intercalation of AgNPs in the DNA helix may prevent the transcription of some genes [89]. Additionally, AgNPs cause the DNA molecule to transition from its relaxed state to its compacted shape, which impairs DNA replication [142]. The first stages of cell division are decreased when AgNPs connect with *S. aureus*, indicating that the interaction of the Ag (+) ions with DNA may play a role in inhibiting cell division and reproduction [143, 144].

## 5. Current Challenges and Future Perspectives

The varying nature of the nanoparticle stability, the absence of its verifiable potential toxicity (inhaling specific nanoparticles may cause gene alterations, allergic reactions, or localized lung inflammation), the development of bacterial resistance to NPs, and the fact that they are difficult to handle in physical form (since particle–particle aggregation occurs due to their small size and large surface area) are the practical challenges of using these nanoparticles in food processing industries and clinical settings. As a result, NPs can pose serious risks to both the environment and human health. Therefore, in vivo studies focusing on the inorganic nanoparticles' antibacterial activity and understanding their unique mechanisms are highly encouraged to directly apply to the host and food processing industries. The in vivo synergistic effects of inorganic nanoparticles with the combination of different antimicrobial agents and recording their biological activities, alternative mechanisms, and potential toxicity are another area to be investigated. Furthermore, the bacterial resistance mechanisms to inorganic nanoparticles are further recommended for study.

## 6. Conclusion

Foodborne diseases are major public health concerns that cause morbidity and mortality across the globe. Inorganic NPs showed effective antibacterial activities against foodborne bacterial pathogens and demonstrated various antibacterial mechanisms. The antibacterial activities of inorganic NPs were greatly affected by the particle size, shape, charge, concentration, type of capping agent, and tested isolates. Cell wall interaction and membrane penetration, ROS production, DNA damage, and protein synthesis inhibition were some of the generalized mechanisms recognized in the current study. This makes inorganic NPs a promising candidate for the development of new antibacterial agents that can combat foodborne bacterial pathogens. However, further research is needed to fully understand the potential toxicity risks and benefits of using inorganic NPs as antibacterial agents.

## Figures and Tables

**Table 1:** Antibacterial activities and mechanisms of inorganic nanoparticles against foodborne bacterial pathogens.

Type of NP	Synthesis method	Characterization method	Size (nm)	Foodborne bacterial pathogens		Antibacterial mechanisms	Main result	References
				G+ bacteria	G− bacteria			
Au	Chemical method	TEM and UV–VIS	11–22	*L. monocytogenes*, *B. cereus*, and *S. aureus*	*S*. Typhimurium, *E. coli* O157:H7, and *P. aeruginosa*	Disruption of the cell wall cell membrane damage	AuNPs were more effective against Gram-negative bacteria as compared to Gram-positives because of the thin peptidoglycan layer in their cell wall	[24]

Ag	Biological method	UV–VIS, XRD, SEM, EDS, TEM, and FTIR	1–10	*B. subtilis*	*K. planticola*, *K. pneumoniae*, *S. nematodiphila*, and *E. coli*	(i) DNA damage (ii) Disruption of the cell wall (iii) Protein oxidation (iv) Interrupting electron transport (v) Formation of reactive oxygen species	Pathogen growth rate decreased at the increased concentration of AgNPs	[25]

ZnO	Biological method	UV–VIS, and HR-TEM	29.7	*S. aureus*	Salmonella spp. *E. coli*	(i) Cellular material leakage (ii) Cell wall and membrane damage (iii) Formation of reactive oxygen species	ZnONP demonstrated effective antibacterial actions against poultry-associated foodborne pathogens	[26]

Ag	Chemical method	UV–VIS, TEM, EDS, SAED, and ICP-MS	15.8 ± 2.2	*S. aureus*	*P. aeruginosa*, *S. enterica*, and *E. coli*	(i) Nucleation and particle growth through surface reduction (ii) Particle growth through coalescence (iii) Particle growth through Ostwald ripening	Reducing the size of NPs in a controlled manner is the key to increasing the effectiveness of their biocidal performance against harmful bacteria	[27]

Ag	Biological method	UV–VIS, XRD, SEM, FTIR, and TGA/DTG	45.26	*B. cereus*, *S. aureus*, *and L. monocytogenes*	*E. coli* and *S*. Typhimurium	Loss of cell wall Damage cell membrane Degradation of enzymes Inactivation of cellular proteins Breakage of DNA	The AgNPs displayed positive antibacterial activity against different foodborne pathogenic bacteria, as well as strong synergistic antibacterial and anticandidal activity with low concentrations of antibiotics and anticandidal agents	[28]

Au	Biological method	TEM and SAXS	8.99	–	*P. shigelloides* and *S. flexneri*	Alterations in the bacterial cells (i) Disruption of the Cell Membrane Structure and integrity Interact directly with DNA	AuNP-GA decreases bacterial pathogenicity by altering the composition of the bacterial membrane compositions	[29]

Au	Biological method	UV–VIS, FE-SEM, EDS, HR-TEM, and FTIR	–	*B. cereus*, *S. aureus*, *and L. monocytogenes*	*E. coli*, *S*. Typhi, and *S. enterica*	(i) Cell membrane damage	Gold nanoparticles synthesized from plant extracts proved to be potent antibacterial agents against food spoilage pathogens	[30]

Se	Biological method	UV–VIS, TEM, XRD, and ATR-FTIR	10–50	*B. cereus*, *E. faecalis*, *and S. aureus*	*E. coli* O157:H7, *S*. Typhimurium, and *S*. Enteritidis	–	The increase in SeNP concentration increases the inhibition effect on the growth of foodborne pathogens	[31]

ZnO	Biological method	DLS, UV–VIS, XRD, FTIR, and SEM	<50	*S. aureus*	*E. coli*	–	*Azadirachta indica*-mediated ZnO-NPs displayed significant antimicrobial activity against foodborne pathogens as compared to Chem-ZnO-NP	[32]

Au	Chemical method	UV–VIS, TEM, FE-SEM, ICP-MS, and XRD	14.7, 45.7, and 31.2	*S. aureus*	*E. coli* and *P. aeruginosa*	Loss of the cell wall Loss of flagella (i) Loss cellular integrity (ii) Loss of cellular matrix	AuNPs demonstrated better antibacterial activity with complete loss of bacterial cells, including nucleic acid and flagella	[33]

Ag	Chemical method	UV–VIS	10	*L. monocytogenes*	*E. coli* O157:H7, *S*. Typhimurium, and *V. parahaemolyticus*	–	AgNPs showed great antibacterial effects on four important foodborne pathogens	[34]

Ag	Biological method	DLS, HR-TEM, ATR-FTIR, XRD, and FE-SEM	∼45	*B. subtilis*, *S. faecalis*, *M. luteus*, and *L. innocua*	*E. coli*	–	AgNPs can be used to control the growth of foodborne pathogens and have potential application in the food packaging industry	[35]

Ag	Biological method	UV–VIS, XRD, FTIR, and HR-TEM	15	*S. aureus*	*E. coli*	(i) Disrupt the cell membrane (ii) Damage the mitochondria (iii) Respiratory enzyme inhibition (iv) Damage the protein structure (v) Damage the nucleic acid	ANE-AgNPs exhibited potent antibacterial efficacy against food-borne pathogens	[36]

MgO	Chemical method	SEM	20	–	Salmonella Stanley *E. coli* O157: H7	(i) Changes in cell morphology (ii) Disruption of normal cell structure and function	MgONPs have shown strong antibacterial activity against important foodborne pathogens	[37]

Se	Biological method	UV–VIS, FE-SEM, FTIR, XRD, HR-TEM, and DLS	–	*S. aureus*	*E. coli S*. Typhi	–	SeNPs mediated by *N. ciliates* exhibited growth inhibitory performance against the pathogenic bacteria	[38]

Ag	Biological method	UV–VIS, SEM, FTIR, and PXRD	6.93	*S. aureus*, *B. subtilis*, *B. cereus*, and *L. monocytogenes*	*E. coli*, *S*. Typhi, and *P. aeruginosa*	(i) Pore creation on the cell and membrane (ii) Cell wall and membrane damage (iii) DNA damage (iv) Enzymatic inactivation (v) Protein denaturation (vi) Production of reactive oxygen species	GC-AgNPs possess excellent antimicrobial properties against six foodborne pathogens	[39]

Ag	Biological method	UV–VIS, FTIR, XRD, and TEM, EDS, DLS	20–25	*B. cereus*, *S. aureus*, and *L. monocytogenes*	*E. coli* and *P. aeruginosa*	–	Green-synthesized AgNPs demonstrated excellent antibacterial activity against foodborne pathogens	[40]

Ag	Biological method	UV–VIS, SEM, AFM, and TEM,XRD	20–100	*B. cereus*, *S. aureus*, and *L. monocytogenes*	*E. coli*, *P. aeruginosa*, and *S*. Enteritidis	(i) Pore creation on the cell and membrane (ii) Cell wall and membrane damage (iii) DNA damage (iv) Inhibiting cell division	Synthesized Jc-AgNps showed significant bactericidal activity against Gram-positive and Gram-negative foodborne bacterial pathogens and were highly effective against *E. coli*	[41]

Ag	Biological method	UV–VIS, XRD, FTIR, and TEM,	4.06	–	*E. coli*, *K. pneumoniae*, *S*. Typhimurium, and *S*. Enteritidis	(i) Formation of free radicals (ii) Penetrate into the cell (iii) Inactivation of proteins	AgNPs exhibit a strong antimicrobial activity against Gram-negative foodborne bacterial pathogens	[42]

Ag	Biological method	UV–VIS, XRD, FE-SEM, FTIR, and EDS DLS	20–30	*S. aureus*	*E. coli*, *S*. Typhimurium, *P. aeruginosa*, and *A. baumannii*	(i) Disrupt the cell wall (ii) Damage the membrane (iii) Leakage of cellular material	The synthesized AgNPs showed excellent antioxidant, antimicrobial, and antibiofilm activity against tested bacterial pathogens	[43]

ZnO	Chemical method	XRD and FE-SEM	10–40	*B. cereus*	*S*. Typhimurium	(i) Pore creation on the cell and membrane (ii) Production of reactive oxygen species	ZnO nanostructures exhibit effective antibacterial activity against foodborne bacterial pathogens	[44]

Ag	Biological method	UV–VIS, DLS, TEM, XRD, and FTIR	21	*S. aureus* (MRSA), *S. epidermis* (MDR), *S. aureus*, *and L. monocytogenes*	*E. coli* O157:H7, *S*. Typhimurium	(i) Disruption of ATP Production and DNA replication (ii) Production of reactive oxygen species (iii) Damage of the membrane	AgNPs demonstrated effective antimicrobial activity against foodborne and other pathogens	[45]

Fe_2_O_3_	Chemical method	FE-SEM, TEM, and DLS	–	*S. aureus*, *B. cereus*, and *L. monocytogenes*	*E. coli*	(i) Pore creation on the cell and membrane (ii) Damage cellular matrix	IONPs showed effective antibacterial activity against selected foodborne pathogens	[46]

ZnO	Chemical method	UV–VIS	110	*B. cereus* and *L. monocytogenes*	*E. coli* O157:H7	–	Natural antimicrobials in combination with nanoparticles effectively inhibit foodborne pathogens by improving the safety of refrigerated meat products	[47]

Ag	Biological method	UV–VIS, TEM, XRD, FTIR, and EDX	10	*L. monocytogenes*	*E. coli*, *K. pneumoniae*, and *P. aeruginosa*	(i) Change membrane permeability (ii) Attack the respiratory chain and cell division machinery (iii) Inactivate the enzymes by producing H_2_O_2_	All pathogenic bacteria were highly inhibited when increasing the concentration of SNPs	[48]

MgO	Chemical method	UV–VIS and SEM	20	–	*C. jejuni*, *E. coli* O157:H7, and *S*. Enteritidis	(i) Alter bacterial cell morphology (ii) Disrupts the membrane structure (iii) Release of reactive oxygen species	MgO nanoparticles have strong antibacterial activity against important foodborne pathogens	[49]

ZnO	Chemical method	SEM	≤50 nm	*B. cereus and S. aureus*	*E. cloacae*, *E. coli*, *E. coli* O157:H7, *P. aeruginosa*, *P. fluorescens*, *S*. Enteritidis, and *S*. Typhimurium	(i) Affected membrane function (ii) Induced lactate dehydrogenase leakage (iii) Generated abnormal cell morphology	ZnONP is an effective and powerful antibacterial agent against Gram-positive and-negative foodborne pathogens	[50]

UV–VIS, ultraviolet–visible spectrophotometer; TEM, transmission electron microscopy; FTIR, Fourier transform infrared; XRD, x-ray diffraction; EDS, energy-dispersive spectroscopy; SEM, scanning electron microscopy; SAED, selected area electron diffraction; ICP-MS, Inductively coupled plasma mass spectrometer; TGA/DTG, thermogravimetric and differential thermogravimetric; SAXS, small angle x-ray scattering; DLS, dynamic light scattering; AFM, atomic force microscopy; EDX, energy dispersive x-ray; HR-TEM, high-resolution transmission electron microscopy; FE-SEM, field emission scanning electron microscopy; ATR-FTIR, attenuated total reflection Fourier transform infrared; PXRD, powder x-ray diffraction.

**Table 2:** Tests of antibacterial efficacy of the inorganic nanoparticles against foodborne bacterial pathogens.

Type of NPs	Source of NPs	Foodborne bacterial pathogens		Concentration	Antibacterial efficacy of nanoparticles			Main factors that influence efficacy	References
					MIC	MBC	ZOI (mm)		
Gold	Chemicals (HAuCl_4_·3H_2_O, CTAB, and MUA)	Gram+	*L. monocytogenes*, *B. cereus*, *and S. aureus*	0.5 mM, using different volumes (20, 40, and 50 *µ*L)	0.39 *µ*L 0.19 *µ*L 0.78 *µ*L	–	11, 12, and 14 12, 13, and 14 –, 11, and 13	(i) Concentration (ii) Tested isolates (iii) Particle size (iv) Particle shape	[24]
		Gram−	*S*. Typhimurium, *E. coli O157:H7*, and *P. aeruginosa*		0.097 *µ*L 0.39 *µ*L 0.39 *µ*L	–	10, 12, and 12 11, 12 and 12 10, 16, and 17		

Silver	Bacteria (*Planomicrobium* sp.)	Gram+	*B. subtilis*	Disc diameter (6 mm) that carried 10 mL from different concentrations (30, 60, and 90 *µ*L) of silver suspension	–	–	17, 19, and 21	(i) Tested isolates (ii) Temperature (iii) Crystal structure (iv) Concentration	[25]
		Gram−	*K. planticola*, *K. pneumoniae*, *S. nematodiphila*, *and E. coli*		–	–	14, 22, and 23 15, 18, and 21 21, 25, and 29 21, 23, and 29		

Zinc oxide	Bacteria (*Lactobacillus plantarum* TA4)	Gram+	*S. aureus*	The diameter (6 mm) carried 100 *µ*L of ZnO NP (at concentrations of 1,000, 2,000, 3,000, 4,000, and 5,000 *µ*g/mL)	30 *µ*g/mL	100 *µ*g/mL	11.33, 12, 15, 16, and 19.67	(i) Particle size (ii) Tested isolates (iii) Concentration (iv) Particle charge	[26]
		Gram−	*Salmonella* spp. and *E. coli*		80 *µ*g/mL 60 *µ*g/mL	160 *µ*g/mL 140 *µ*g/mL	8, 9.33, 10.67,12, and 12.33 8, 9, 10.33, 11, and 12		

Silver	Chemicals (AgNO_3_, C_2_H_6_O_2_, and polyethylene glycols)	Gram+	*S. aureus*	Silver colloidal solution (150 mg/mL) was serially diluted in a 96-well microtiter plate	4.69 mg/mL	–	–	(i) Particle size (ii) Tested isolates (iii) Concentration	[27]
		Gram−	*P. aeruginosa*, *S. enterica*, *and E. coli*		2.34 mg/mL 2.34 mg/mL 1.19 mg/mL	–	–		

Silver	Plants (*Zea mays* L.)	Gram+	*B. cereus*, *S. aureus*, *and L. monocytogenes*	6 mm-diameter paper disks containing 50 *µ*g of AgNPs/disk were used for the assay	25 *µ*g/mL 12.5 *µ*g/mL 25 *µ*g/mL	50 *µ*g/mL 25 *µ*g/mL 50 *µ*g/mL	11.39 ± 1.2 11.57 ± 0.25 9.26 ± 0.31	(i) Particle size (ii) Shape (iii) Tested isolates (iv) Concentration	[28]
		Gram−	*E. coli* and *S*. Typhimurium		50 *µ*g/mL 50 *µ*g/mL	100 *µ*g/mL 100 *µ*g/mL	10.55 ± 0.27 11.22 ± 0.38		

Gold	Plant products (gallic acid)	Gram+	–	The 6 mm diameter carried 100 *µ*L of various concentrations of AuNP–GA (at 50, 30, and 10 mM)	–	–	–	(i) Tested isolates (ii) Concentration	[29]
		Gram−	*P. shigelloides and S. flexneri*		110 mM 50 mM	–	–		

Gold	Plants (*Cucurbita pepo* and *Monardella crispa* leaves)	Gram+	*B. cereus*, *S. aureus*, *and L. monocytogenes*	6 mm-diameter well was added with 40 *µ*L of 800 *µ*g/ml concentration of gold nanoparticles			11, and 11 11, and 12–, and 12	(i) Shape (ii) Size (iii) Tested isolates (iv) Concentration	[30]
		Gram−	*E. coli*, *S*. Typhi, and *S. enterica*				12, and 11 12, and 12 12, and 12		

Selenium	Bacteria (isolated from food wastes)	Gram+	*B. cereus*, *E. faecalis*, *and S. aureus*	6 mm-diameter well was added with 250 *µ*L of various concentrations (0, 10, 15, 20, 25, 30, 35, and 40 *µ*g/mL) SeNPs.	25 *µ*g/mL 25 *µ*g/mL 25 *µ*g/mL	–	–	(i) Particle size (ii) Concentration	[31]
		Gram−	*E. coli* O157:H7, *S*. Typhimurium, *and S*. Enteritidis		25 *µ*g/mL 25 *µ*g/mL 25 *µ*g/mL	–	–		

Zinc oxide	Plants (*Azadirachta indica* leaf)	Gram+	*S. aureus*	6 mm-diameter paper disks containing 1 mg/mL ZnO-NP/disk were used in various concentrations (2,3,4,8,10,15,20,24, and 32 *µ*g/mL)	18 mg/mL	–	12.6 ± 0.21, 19 ± 0.39, 22 ± 0.33, 29 ± 0.43, 27 ± 0.37, 35 ± 0.37, 31 ± 0.39, 32 ± 0.43, and 45 ± 0.37	(i) Concentration (ii) Capping/stabilizing agent	[32]
		Gram−	*E. coli*		20 mg/mL	–	11 ± 0.43, 16.5 ± 0.37, 17 ± 0.36, 22 ± 0.38, 24 ± 0.47, 34 ± 0.46, 30 ± 0.38, 36 ± 0.39, and 48.5 ± 0.38		

Gold	Chemicals (NaBH_4_, CTAB, and CTAC)	Gram+	*S. aureus*	6 mm-diameter paper disks containing 7.7677 *µ*g/L of AuNPs/disk were used at a concentration of (80 *µ*g/mL)	0.4 *µ*g/mL	–	16.5	(i) Particle shape (ii) Particle size (iii) Concentration	[33]
		Gram−	*E. coli and P. aeruginosa*		0.4 *µ*g/mL 0.4 *µ*g/mL	–	18.5 20.5		

Silver	Commercialized	Gram+	*L. monocytogenes*	Two-fold serial dilutions of nanosilver solution were prepared in sterile 96-well plates in the range of 0.78–100 *µ*g/mL	6.25 *µ*g/mL	6.25 *µ*g/mL	–	(i) Particle size (ii) Concentration (iii) Particle shape (iv) Tested isolates	[34]
		Gram−	*E. coli* O157:H7, *S*. Typhimurium, *and V. parahaemolyticus*		3.12 *µ*g/mL 3.12 *µ*g/mL 3.12 *µ*g/mL	6.25 *µ*g/mL 6.25 *µ*g/mL 6.25 *µ*g/mL	–		

Silver	Fungi (*Ganoderma sessiliforme*)	Gram+	*B. subtilis*, *S. faecalis*, *L. innocua*, *and M. luteus*	The 5 mm diameter and 2.5 mm deep well was filled with 50 *µ*L of 1 mg/mLconcentration of AgNP suspension	–	–	20 ± 1.00 16 ± 1.00 22 ± 1.15 21 ± 1.15	(i) Particle size (ii) Particle charge (iii) Tested isolates (iv) Concentration (v) Capping/stabilizing agent	[35]
		Gram−	*E. coli*		–	–	11 ± 0.50		

Silver	Plant (*Argyreia nervosa* leaf)	Gram+	*S. aureus*	6 mm diameter disc loaded with 25 *µ*L of various concentrations (10, 25, and 50 *µ*g/mL) of ANE-AgNPs. For MIC, serial twofold dilutions of ANE-AgNPs were prepared in sterile 96-well plates at 5, 10, 20, 30, 40, 50, 75, and 100 *µ*g/mL	60 *µ*g/mL	–	12.5 ± 0.18, 15.2 ± 0.16, and 17.5 ± 0.18	(i) Particle size (ii) Tested isolates (iii) Concentration (iv) Capping/stabilizing agent	[36]
		Gram−	*E. coli*		40 *µ*g/mL	–	17.5 ± 0.21, 20.2 ± 0.28, and 22.1 ± 0.19		

Silver	Animal (goat colostrum)	Gram+	*S. aureus and B. cereus*	6 mm-diameter paper disks containing 5 mg/mL, 10 mg/mL of GC-AgNPs/disk were used for the assay. For MIC, 100 *μ*L of GC-AgNP (200–1 *μ*g/mL) was poured into the microtiter	– 30 ± 0.1 *μ*g/mL	– 30 ± 0.4 *μ*g/mL	18.97 ± 0.47, and 20.65 ± 0.05 15.08 ± 0.25, and 25.78 ± 0.38	(i) Tested isolates (ii) Concentration	[39]
		Gram−	*E. coli and P. aeruginosa*		15 ± 0.1 *μ*g/mL –	15 ± 0.1 *μ*g/mL –	23 ± 1.92, and 29.27 ± 0.75 40.27 ± 0.56, and 38.03 ± 0.34		

Silver	Plants (pu-erh tea leaves)	Gram+	–	A 6 mm diameter well was added with 10 *µ*L of 1 mM concentration of AgNPs. For MIC, 100 *µ*L of the synthesized AgNP stock solution (500 *µ*g/mL) was added and diluted twofold.	–	–	–	(i) Tested isolates (ii) Concentration (iii) Particle size (iv) Particle shape (v) Antibiotic synergy	[42]
		Gram−	*E. coli*, *K. pneumoniae*, *S*. Typhimurium, *and S*. Enteritidis		7.8 *µ*g/mL 3.9 *µ*g/mL 3.9 *µ*g/mL 3.9 *µ*g/mL	7.8 *µ*g/mL 3.9 *µ*g/mL 7.8 *µ*g/mL 3.9 *µ*g/mL	15 10 20 20		

Silver	Plants (*Crocus sativus* L. fower (saffron))	Gram+	*S. aureus*	8 mm wells were filled with 50 *µ*L of AgNP suspensions in various concentrations of 80, 160, 320, 640, 1,280, and 2,560 (mg/L).	320 mg/L	320 mg/L	–, –, –, 11, 13, and 14	(i) Tested isolates (ii) Concentration (iii) Particle size (iv) Particle shape	[43]
		Gram−	*E. coli*, *S*. Typhimurium, *P. aeruginosa*, *and A. baumannii*		640 mg/L 640 mg/L 320 mg/L 640 mg/L	640 mg/L 1,280 mg/L 640 mg/L 1,280 mg/L	–, 9, 10, 11, 13, and 14 –, –, –, – 11, and 12 –, –, 10, 12, 13, and 15 –, 8, 8, 10, 12, and 13		

Zinc oxide	Chemicals ((CH_3_ COO)_2_Zn.·2H_2_O, NH_2_.CO.NH_2_, and C₂H₆O₂)	Gram+	*B. cereus*	MIC and MBC were determined by incubation of test bacteria with different concentrations of ZnO powder in the range of 15 *µ*g - 1,000 *µ*g/mL	125 *µ*g/mL	250 *µ*g/mL	–	(i) Tested isolates (ii) Concentration	[44]
		Gram−	*S*. Typhimurium		250 *µ*g/mL	500 *µ*g/mL	–		

Silver	Bacteria (*Streptomyces atroverins*, strain Askar-SH50)	Gram+	*S. aureus* (MRSA), *S. epidermis* (MDR), *S. aureus*, *and L. monocytogenes*	Paper disks of AgNPs (105 ppm) with different concentrations (250, 125, 62.50, 31.25, 15.63, 7.81, 3.90, 1.95, 0.98, 0.49, 0.24 and 0.12 *μ*g/mL)	53.5 *µ*g/mL 26.75 *µ*g/mL 53.5 *µ*g/mL 26.75 *µ*g/mL	–	18 ± 0.52 21 ± 0.40 15 ± 0.36 18 ± 0.54	(i) Tested isolates (ii) Concentration (iii) Particle size	[45]
		Gram−	*E. coli O157:H7 S*. Typhimurium		62.5 *µ*g/mL 53.5 *µ*g/mL	–	17 ± 0.32 18 ± 0.35		

Iron oxide	Chemicals (C_18_H_37_N, Na_3_C_6_H_5_O_7_, C₂H₆O₂, and FeCl_3_·6H_2_O)	Gram+	*S. aureus*, *B. cereus*, *and L. monocytogenes*	For MIC, serial two-fold dilution (25, 12.5, 6.25, 3.125, 1.56, 0.78 and 0.39 *µ*g/mL) of IONPs	12.5 *µ*g/mL >25 *µ*g/mL >25 *µ*g/mL	–	–	(i) Particle size (ii) Tested isolates (iii) Concentration	[46]
		Gram−	*E. coli*		6.25 *µ*g/mL	–	–		

Zinc oxide	Commercialized	Gram+	*B. cereus and S. aureus*	6 mm paper disks filled with 10 *µ*L of ZnO stock solution. For MIC, 100 *µ*L of a ZnO suspension were done by serial dilutions to give a final ZnO concentration in the range of 1–200 mM	7 mM 10 mM	–	31 ± 1.4 35 ± 1.6	(i) Particle size (ii) Tested isolates (iii) Concentration (iv) Particle charge	[50]
		Gram−	*E. cloacae*, *E. coli*, *E. coli* O157:H7, *P. aeruginosa*, *P. fluorescens*, *S*. Enteritidis, and *S*. Typhimurium		21 mM 16 mM 17 mM 26 mM 24 mM 20 mM 22 mM	–	19 ± 1.4 21 ± 1.3 18 ± 1.1 17 ± 1.2 18 ± 1.1 22 ± 1.2 21 ± 1.4		

CTAB, cetrimonium bromide; MUA, meta uredo aniline; HAuCl_4_·3H_2_O, gold (iii) chloride trihydrate; AgNO_3_, silver nitrate; C_2_H_6_O_2_, ethylene glycol; NaBH_4_, sodium borohydride; CTAC, cetrimonium chloride; CH_3_ COO_2_Zn.2H_2_O, zinc acetate dihydrate; NH_2_.CO.NH_2_, urea; C_18_H_37_N, oleylamine; Na_3_C_6_H_5_O_7_, sodium citrate; FeCl_3_·6H_2_O, iron (iii) chloride hexahydrate.

**Table 3:** Factors affecting the antibacterial efficacies of inorganic nanoparticles.

No	Influencing factors	Main effects	Scientific reasons/mechanisms	References
1.	Particle origin	As compared to incidental and natural sources, engineered NPs antibacterial efficacy is increased by creating the desired size, shape, porosity, and large surface area for reactions	It follows a simple preparation process and makes the NPs have good optical, electrical, catalytic, and magnetic behavior, along with increasing their chemical and mechanical stability	[52]

2.	Bacterial Gram-type	The antibacterial activities of NPs were higher among Gram-negative bacteria than Gram-positives	The cell walls of Gram-positive bacteria are composed of ∼20–80 nm peptidoglycan, which is comparatively thicker than ∼7–8 nm peptidoglycan found in Gram-negative bacteria	[11]

3.	Particle size	Small-sized NPs have the greatest antimicrobial effect in comparison to larger ones	Smaller particles have more surface area for releasing ions which enhances the protein binding capabilities and the easily flow of ions through the pores in the bacterial membrane and readily reach the bacteria	[53]

4.	Particle shape	Spherical-shaped NPs have the highest bactericidal effect as compared to the other NP shapes	Facet (111) has a high atomic density, which improves the ability of NPs to release more ions, which can bind to components that contain sulfur of the bacteria	[54]

5.	Particle charge	The positively charged, neutral, and negatively charged AgNPs, respectively, showed the highest, intermediate, and least level of antibacterial activity against the organisms tested	NPs with positive charges interact with negative charges on bacteria cell membranes, disrupting their cell walls and surface proteins and ultimately causing cell death	[55]

6.	Concentration	The zone of inhibition (ZOI) is increased with increasing NPs concentration	The higher concentration of NPs releases more ions, which in turn increase cellular oxidative stress, producing higher antibacterial activity than their counterparts (low concentrations)	[56]

7.	Capping/stablizing agents	NPs with various capping agents display various antibacterial properties	When varying the reducing and protective agents, the particle size of NPs also varied from extremely small to larger and consequently affected the antimicrobial efficacy of NPs either positively or negatively	[57]

8.	Reaction conditions during synthesis of NPs	(i) pH (when the pH increases the size of the produced nanoparticle decreases). (ii) Temperature (when the temperature decreases the particle size also decreases). (iii) Pressure (both the shape and size of the synthesized nanoparticle depends on the amount of pressure we applied to the reaction medium). (iv) Concentration (optimum concentration is required for homogenous particle synthesis) (v) Reaction or incubation time (the size and shape of produced nanoparticle varies with varying reaction or incubation time)	Green synthesis (biological methods) enables advancement over physical (usually require very high time, energy, pressure, and temperature conditions to synthesize the NPs) and chemical (high toxicity, expensive) methods with simple, ecological suitability, easily scaled up for large-scale synthesis and minimize nanoparticle preparation costs while increasing nanoparticle`s antibacterial efficacy	[58]

## Data Availability

No new data were created or analyzed during the review process, but only combined those single studies into one and provide complete information for readers and stakeholders.

## Abbreviations

AgNPs: Silver nanoparticles
AuNPs: Gold nanoparticles
CuO-NPs: Copper oxide nanoparticle
IONPs: Iron oxide nanoparticles
MBC: Minimum bactericidal concentration
MgO-NPs: Magnesium oxide nanoparticles
MIC: Minimum inhibitory concentration
NPs: Nanoparticles
RISMA: Preferred reporting items for systematic reviews and meta-analyses
ROS: Reactive oxygen species
SeNPs: Selenium nanoparticles
ZnO-NPs: Zinc oxide nanoparticles
ZOI: Zone of inhibition.
